# Patient Preferences and Cosmetic Outcomes Following Destructive Treatments for Non-facial Basal Cell Carcinoma: A Mixed Methods Study

**DOI:** 10.2340/actadv.v105.41325

**Published:** 2025-02-12

**Authors:** Eva BACKMAN, Birgit HECKEMANN, Martin GILLSTEDT, Sam POLESIE, John PAOLI

**Affiliations:** 1 Department of Dermatology and Venereology, Institute of Clinical Sciences, Sahlgrenska Academy, University of Gothenburg, Gothenburg; 2 Region Västra Götaland, Sahlgrenska University Hospital, Department of Dermatology and Venereology, Gothenburg; 3 Institute of Health and Care Sciences, Sahlgrenska Academy, University of Gothenburg, Gothenburg; 4 Region Västra Götaland, Sahlgrenska University Hospital, Department of Anesthesiology and Intensive Care Medicine/Pain Centre, Gothenburg, Sweden

**Keywords:** basal cell carcinoma, cryosurgery, curettage, patient preference, patient satisfaction, treatment outcome

## Abstract

The high prevalence of basal cell carcinoma (BCC) entails a substantial burden on healthcare systems. Interest in non-surgical treatment methods for low-risk BCCs, including destructive treatments, is increasing. Dermatologists often highlight suboptimal cosmetic outcomes as drawbacks of destructive treatments, also for non-facial lesions. Patient perspectives regarding scarring and cosmetic outcomes in relation to other relevant factors are largely unknown, yet important to consider in shared decision-making when choosing treatment. This study investigates patient perceptions of scarring following destructive treatments and explores important factors in treatment decisions for non-facial BCCs. Through a mixed-methods design, cosmetic outcomes, patient satisfaction, scarring concerns, and treatment preferences were evaluated within an ongoing randomized clinical trial on destructive treatments for low-risk BCCs. Overall, 157 patients with 425 non-facial scars were assessed. Most patients were not concerned about scar appearance, highlighting a discrepancy compared with dermatologists’ general concerns regarding inferior cosmetic outcome. Instead, when opting for specific treatments, patients listed clearance rates as the most important factor, followed by convenience and time consumption. We believe the results are important both in the context of patient-centred care and in “choosing wisely” when deciding between BCC treatments.

## SIGNIFICANCE

Basal cell carcinomas (BCCs) significantly impact healthcare costs due to high prevalence. Non-surgical treatments, including destructive treatments, for low-risk basal cell carcinomas are gaining popularity. Yet, patient perspectives on scarring and treatment preferences remain underexplored. This study reveals that patients with non-facial basal cell carcinomas prioritize treatment effectiveness, convenience, and time consumption over scarring concerns. Evaluating 157 patients with 425 non-facial scars, the absolute majority of patients were satisfied with cosmetic outcomes despite dermatologists’ general concerns regarding inferior cosmetic outcome. This underscores the importance of aligning treatment decisions with patient preferences, promoting patient-centred care and shared decision-making in basal cell carcinoma management.

Basal cell carcinoma (BCC) is the most common tumour among fair-skinned populations. Its diagnosis and treatment add pressure to limited healthcare resources (1–5). Low-risk BCCs, i.e., primary lesions outside the facial area with superficial or nodular growth patterns, make up a substantial proportion of these tumours (6–8). Besides surgery, several treatment options are available for low-risk BCCs (9, 10). These include topical treatments such as 5-fluorouracil, imiquimod, and photodynamic therapy (PDT) (11). Additionally, destructive treatments including curettage, electrodesiccation, and cryosurgery are possible options for both superficial and nodular BCCs (12–16). A disadvantage of destructive treatments is scarring, often presenting as a hypopigmented area with or without atrophy or induration. The inferior cosmetic outcome of destructive treatments is often highlighted in comparison with PDT (even for non-facial low-risk lesions) and with surgery (17–19). However, few studies have investigated patient perceptions regarding the resulting scars (i.e., relationships between cosmetic outcome, patient satisfaction, and anatomical site) and little is known about the relevant factors regarding patient treatment preferences (20).

The aim of this study was to evaluate the cosmetic outcome and its correlation with patient satisfaction after destructive treatments for non-facial BCCs. Further, the study explored factors of importance to patients when deciding upon treatments, e.g., clearance rate, cosmetic outcome, the time required for treatments, the healing process, and cost considerations associated with different treatments.

## PATIENTS AND METHODS

### Context

**Fig. 1:**
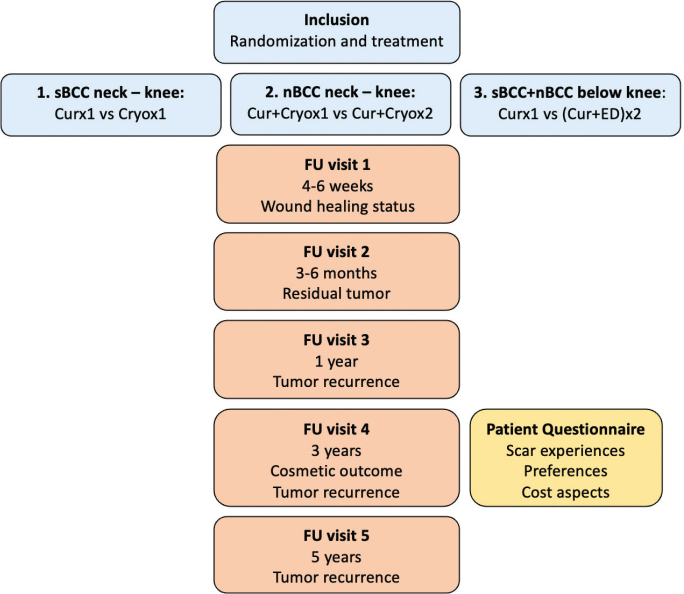
**Overview of the randomized controlled trial with performed procedures/evaluations.** Cur: curettage; cryo: cryosurgery; ED: electrodesiccation; FU: follow-up visit; nBCC: BCC with nodular features; sBCC: superficial BCC; vs: versus.

This investigation is part of a randomized controlled trial (RCT) on destructive treatments of non-facial low-risk BCCs conducted at the Department of Dermatology and Venereology, Sahlgrenska University Hospital in Gothenburg, Sweden (available at https://www.researchweb.org/is/vgr/project/259511) (13, 16). Patients were recruited between November 2017 and March 2022 and will be followed for 5 years (**Fig. 1**). Superficial BCCs between the neck and knees were randomized to either curettage alone or cryosurgery in 1 freeze–thaw cycle, nodular BCCs between the neck and knees were randomized to curettage followed by 1 or 2 freeze–thaw cycles, and finally superficial and nodular BCCs below the knees were randomized to either curettage alone or curettage plus electrodesiccation repeated twice.

### Study design, setting, and participants

This study on cosmetic outcome and patient preferences has a mixed-methods design (21). All patients who had attended their 3-year follow-up visit in the RCT were eligible to participate in the study. At the 3-year follow-up, both the patient and the dermatologist assessed the cosmetic outcome. Patients also completed a survey regarding their perspectives on their scars, previous BCC treatments, treatment preferences, and cost considerations in a tax-funded healthcare system. This was a written questionnaire, posted via mail to all patients who had completed their 3-year follow-up. Quantitative data constitute the predominant part of the analysis, while qualitative data have a supporting role to provide depth of understanding (21).

### Tools and data collection

*Quantitative assessment of cosmetic outcome (ACO).* Scars were evaluated by the patient and the dermatologist using a numeric rating scale (NRS), ranging from 1–10, developed within the research group (Appendix S1). Initially, we considered using the Patient and Observer Scar Assessment Scale (POSAS, version 2 [in Swedish]) (22). However, a previous report concluded that the reproducibility was lower than expected for non-surgical scars following BCC treatments (23). Further, when we performed pilot testing of the Patient Scar Assessment Scale (PSAS), we concluded that it was too comprehensive and, most importantly, failed to address our research questions regarding patient satisfaction and concerns about scarring. All patients who had attended their 3-year follow-up visit as of 13 February 2023, completed the ACO (*n* = 157).

A research nurse invited patients to rate their scar(s) from 1 to 10 using the following 3 questions:

1. How much do you care about the scar? (1, not at all; 10, very concerned),
2. How satisfied are you with the scar? (1, very satisfied; 10, very unsatisfied), and
3. What is your overall opinion of the scar appearance, as compared with your normal skin? (1, normal skin; 10, very different).

A dermatologist (1 of 7 involved in the study treatment and /or follow-up visits) rated the scars by 3 different parameters (vascularity, pigmentation, and thickness) and provided their overall opinion of each scar using a scale from 1 to 10 (1, normal skin; 10, worst scar imaginable).

*Questionnaire on patients’ perspectives on scars, treatments, preferences, and cost (STPC).* A questionnaire with 14 open- and closed-ended questions (Q1–14) was designed to gain insights on patients’ perspectives on scars, experiences from earlier treatments, treatment preferences, and cost considerations. The questionnaire (Appendix S2) covered 6 main areas:

1. Experiences with the scars (Q1–4).
2. Whether patients recalled being informed about the risk of scarring (Q5–6).
3. Current and previous BCC treatment experiences (Q7–10).
4. Possible future treatment preference for superficial BCC (sBCC) (Q11–12).
5. Ranking factors of importance when deciding upon treatment for BCC (Q13).
6. Patient opinions on cost considerations when choosing treatment methods for sBCCs in a tax-funded healthcare system (Q14).

The questionnaire was reviewed within the research group, which included a senior researcher with experience in qualitative research (BH) and underwent pilot testing with 2 patients to ensure that the questions were understandable and relevant. The questionnaire was distributed to eligible patients in the RCT who had completed or were scheduled for their 3-year follow-up before 1 March 2023, and remained enrolled in the study (*n* = 155). The patients received the questionnaire by mail, and non-respondents were sent a reminder after 3 weeks. Data collection occurred from 1 February to 17 April 2023.

### Statistical analysis

Quantitative data from the ACO questionnaire and certain sections of the STPC questionnaire were analysed to generate descriptive statistics. The Wilcoxon rank-sum test, Kruskal–Wallis test, and Fisher’s exact test were used to test for significant differences between groups. The presence of correlations between satisfaction, concern for the scar, sex, and age was explored using the Spearman correlation test. The significance level was set at *p* < 0.05, and all tests were two-sided. Statistical calculations were performed using R, version 3.5.3. (R Foundation for Statistical Computing, Vienna, Austria).

For the free-text answers from the STPC questionnaire, verbatim responses were transferred to a Microsoft Excel (Microsoft, Redmond, WA, USA) file. Quantitative and qualitative content analyses were conducted according to Krippendorff’s methodology (24). The answers were inductively coded and categorized by their manifest content. This part was conducted by EB and was subsequently reviewed by BH. Any discrepancies were discussed until consensus was achieved.

## RESULTS

### Demographics

Overall, 157 patients with 425 scars were included in the ACO analysis. The majority of patients were male (*n* = 96, 61.1%) and males had a higher proportion of scars (*n* = 299, 70.4%). The median age at study inclusion was 70.2 years, with females being slightly younger than males (**Table I**).

**Table I:** Patient and tumour characteristics for the quantitative assessment of cosmetic outcome (ACO) questionnaire at the 3-year follow-up visit

Variable	Females	Males	Total	*p*-value
Patients, *n* (%)	61 (38.9)	96 (61.1))	157 (100)	
Lesions, *n* (%)	126 (29.6)	299(70.4)	425 (100)	
Median age at inclusion (range)	68.3 (30.5–83.0)	72 (29.3–87)	70.2 (29.3–87.0)	0.006
Lesions per age group (years)				
≤50	10 (7.9)	11(3.7)	21 (4.9)	
51–60	25 (19.8)	24 (8.0)	49 (11.5)	
61–70	36 (28.6)	49 (16.4)	85 (20.0)	< 0.0001
71–80	52 (41.3)	168 (56.2)	220 (51.8)	
>80	3 (2.4)	47 (15.7)	50 (11.8)	
Lesions in patients ≤70 years	71 (56.3)	84 (28.1)	155 (36.5)	< 0.0001
Lesions in patients >70 years	55 (43.7)	215 (71.9)	270 (63.5)	
Lesions per location, *n* (%)				
Neck	1 (0.8)	15 (5.0)	16 (3.8)	
Trunk	71 (56.3)	173 (59.7)	244 (57.4)	
Upper limbs	15 (11.9)	41 (13.7)	56 (13.2)	0.11
Thigh	10 (7.9)	13 (4.3)	23 (5.4)	
Below knees	29 (23.0)	57 (19.1)	86 (20.2)	
Treatments				
Cur & Cryo x1 (nBCC above knee)	22 (17.5)	47 (15.7)	69 (16.2)	
Cur & Cryo x2 (nBCC above knee)	20 (15.9)	50 (16.7)	70 (16.5)	
Cur x1 (sBCCs above knee)	27 (21.4)	76 (25.4)	103 (24.2)	0.72
Cryo x1 (sBCCs above knee)	28 (22.2)	69 (23.1)	97 (22.8)	
Cur x1 (s+nBCCs below knee)	12 (9.5)	31 (10.4)	43 (10.1)	
Cur & ED x2 (s+nBCCs below knee)	17 (13.5)	26 (8.7)	43 (10.1)	

*P*-values correspond to differences in sex distribution across the respective variable categories; *n*: number; cur: curettage; sBCC: superficial BCC; cryo: cryosurgery; nBCC: nodular BCC; ED: electrodesiccation.

The STPC questionnaire was distributed to 155 patients and 135 were returned, yielding a response rate of 87.1%. All questionnaires were included in the analysis, regardless of whether they were fully or partly completed. The median age of the respondents at the time of questionnaire completion was 74.8 years (range 34.5–91.5 years) with 59.3% (*n* = 80) being male. The total number of scars represented was 372. Among 130 respondents, 49% (*n* = 64, missing data *n* = 5) had prior experience with alternative treatments for BCCs, including PDT, surgery (traditional or Mohs surgery), other destructive treatments, and imiquimod.

### Quantitative assessments of scars (ACO)

*Cosmetic outcome: patient and dermatologist rating.* For the majority of scars (*n* = 341, 82.6%), patients reported that they did not care at all about the appearance of their scars (NRS 1–2), with a mean overall NRS score of 1.7. Further, patients’ overall satisfaction with their scars was high, with a mean NRS score of 2.2. The mean NRS score for the overall opinion of the scars compared with normal skin was 3.6 (more different from normal skin), indicating that the evaluation of scar cosmesis was not consistent with their satisfaction with the scars. The dermatologist’s rating of the overall opinion of the scars compared with normal skin was in the same range as the ratings of patients, with a mean NRS score of 3.1 (**Table II**).

**Table II:** Results of the quantitative assessment of cosmetic outcome (ACO) questionnaire conducted by both the patient and the dermatologist at the 3-year follow-up visit (missing data excluded)

Factor	Numeric rating scale score							
	1–2	3–4	5–6	7–8	9–10	Median (range)	Mean (SD)	Missing data
Patients								
Care about scar, *n (%)*	341 (82.6)	42 (10.2)	20 (4.8)	8 (1.9)	2 (0.5)	1 (1–9)	1.7 (1.5)	12
Overall satisfaction with scar, *n* (%)	296 (71.7)	68 (16.5)	32 (7.7)	10 (2.4)	7 (1.7)	1 (1–10)	2.2 (1.9)	12
Overall scar appearance, *n* (%)	115 (28.2)	168 (41.2)	78 (19.1)	42 (10.3)	5 (1.2)	3 (1–10)	3.6 (2.0)	17
Dermatologists								
Overall scar appearance, *n* (%)	114 (27.9)	265 (64.8)	27 (6.6)	3 (0.7)	0 (0)	3 (1–8)	3.1 (1.0)	16

Lower scores indicate a more positive evaluation of the scars; *n:* number; SD: standard deviation.

**Fig. 2:**
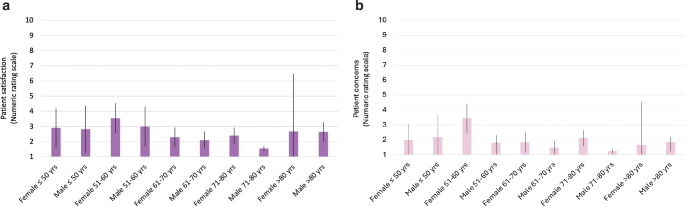
**(A) Patient satisfaction with the scars and (B) patient concerns about the scars (mean and 95% confidence interval), stratified by sex and age groups.** Lower scores indicate a more positive evaluation of the scars.

*Differences in satisfaction and concern about scars between sex and age groups.* Male patients cared slightly less about their scars than female patients, with a mean NRS score of 1.5 (95% confidence interval [CI] 1.3–1.6) vs 2.3 (95% CI 1.9–2.7) (*p* < 0.0001). Male patients were also slightly more satisfied than female patients with a mean NRS score of 2.0 (95% CI 1.8–2–2) vs 2.6 (95% CI 2.3–3.0) (*p* < 0.0001) (Table SI). Furthermore, there was a trend (not significant) indicating that males in the age group of 71 to 80 years, who had the majority of assessed scars, were more satisfied and also cared less about the appearance of their scars than males and females in other age groups. Females in the age group 51 to 60 years reported being less satisfied and were more concerned about their scars (not significant) (**Fig. 2**). There were no statistically significant differences in patients’ overall satisfaction with scar appearance following the different destructive treatment protocols (*p* = 0.44) nor for anatomical sites (*p* = 0.081).

### Qualitative evaluation (STPC questionnaire)

*Experience and satisfaction with scars (STPC areas 1–3).* When asked about their experiences with their scars and what factors influenced their experiences, 95.6% (*n* = 129) of patients reported having no problems or hardly ever thinking about their scars in everyday life. Five female respondents reported dissatisfaction with having a hypopigmented scar, due to its location on the chest (*n* = 3) or on the lower leg (*n* = 2), while one male had developed an itchy keloid on the shoulder.

The majority (*n* = 111, 84.1%) did not feel that their experiences had changed over time, while 13 respondents (9.8%) felt they cared less over time, as the scars had become less visible. One respondent (0.76%) reported caring more after 3 years than initially after treatment when she was above all satisfied with having the BCC removed. Another 7 respondents (5.3%) were unsure and 3 did not reply (**Table III**).

**Table III:** Patients’ experiences with scars and treatment preferences

Variable	Result	Quotes
Satisfaction	Satisfied: *n* = 129	“Visible but not disturbing, don’t think about them”
Non-respondents: *n* = 0		“Age, I might have found the scars bothersome if I were much younger”
		“Glad BCC is gone, don’t care about scar”
		“Have so many other skin lesions”
		“Difference between scars on the body and on the face”
	Dissatisfied: *n* = 6	“Lower leg – ugly scar”
		“The scar on my chest looks ugly, a white spot that is visible and it disturbs me”
Experience/attitude towards scar	Change over time: *n* = 14	“It took time for the scar to subside, at first I thought it would remain
Non-respondents: *n* = 3		elevated”
		“Over time, the scars have faded more and more”
		“Maybe the scars bother me a bit more now. Initially, I was just happy to have the basal cell carcinoma removed”
	No change over time: *n* = 111	
	Unsure: *n* = 7	
Informed about scar	Yes: *n* = 88	“The cosmesis is better than expected”
Non-respondents: *n* = 3		“Rather hyperpigmentation than hypopigmentation” (lower leg)
		“The information was correct”
	No: *n* = 12	
	Do not remember *n* = 32	
Experience with other treatments	Yes: *n* = 64	“All different treatments have been OK”
Non-respondents: *n* = 5		“Destructive treatment is very quick and convenient”
		“PDT was very painful”
		“Surgery was a more difficult experience”
		“SDL-PDT was very convenient but less effective, motivated with more aggressive treatment”
		“PDT gave no scars”
		“More superficial wounds with other treatments”
	No: *n* = 66	
Choice of treatment	Destructive treatment: *n* = 110	“Clearance rate is more important than scar”
Non-respondents: *n* = 15		“Too time consuming” (PDT and cream)
		“Don’t care about scar, age”
		“Cream seems more complicated and I have tried PDT before”
	PDT/imiquimod: *n* = 10	“If exposed area cream or PDT, otherwise destructive treatment”
		“Treatment at home and if not successful, destructive treatment”
Most important factor	Clearance rate: *n* = 83	No comments
Non-respondents: *n* = 12	Time for treatment: *n* = 1	“Treatment at doctor’s first visit is important”
Incomplete answers: *n* = 37	Time for wound to heal: *n* = 1	No comment
	Cosmesis: *n* = 1	“Scar on face”
Cost aspects	Patient’s preference prioritized: *n* = 55	“All BCC treatments are cheap compared to other procedures in healthcare”
Non-respondents: *n* = 26		“Patients may have different opinions about scars which might be important to consider”
	Cost aspects prioritized: *n* = 34	“Most effective treatment with lowest risk of relapse is best also from a socioeconomic perspective”
		“Cost and in dialogue with patient”
	No consideration of cost: *n* = 20	No comments

Data including quotes from the scars, treatments, preferences, and cost (STPC) questionnaire; *n*: numbers; PDT: photodynamic treatment; SDL-PDT: simulated daylight PDT.

**Fig. 3:**
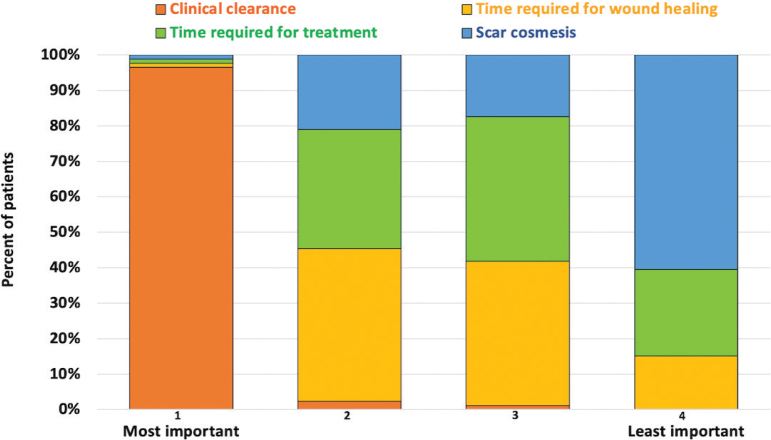
Patients’ ranking of factors of importance when choosing treatment methods for basal cell carcinoma. 1, most important; 2, second most important; 3, third most important; 4, least important.

*Important factors when choosing a treatment method (STPC areas 4–5).* In total, 86 patients (63.4%) gave complete answers when asked to rank the factors of greatest (1) to least (4) importance to them when treating a BCC. The results based on all complete answers (1 to 4) are presented in **Fig. 3** and illustrate that the clearance rate was of the greatest importance for the majority of respondents (96.5%), while scar cosmesis was of the least importance. Time required for treatment as well as time required for wound healing ranked as being of intermediate importance. Another 37 patients (27.4%) gave incomplete answers. Among the 123 patients who gave either complete or incomplete answers, 118 (95.9%) rated the clearance rate as the most important factor.

When asked specifically about a hypothetical future treatment preference for sBCC (see Appendix S2), 91.7% (*n* = 110) favoured destructive treatment, while 8.3% (*n* = 10) preferred PDT or imiquimod to avoid scarring despite a higher risk of recurrence (15 patients did not respond). Patient sex (*p* = 0.71) and previous experience of BCC treatments (*p* = 0.094) showed no significant association with treatment preferences.

*Cost aspects for treatments in a tax-funded healthcare system (STPC area 6).* Of 109 respondents, 50% (*n* = 55) expressed the view that a patient’s own preference should be prioritized over the cost of the treatment, while 31% (*n* = 34) thought the cost should be considered to a great extent. The remaining 18% (*n* = 20) thought that costs should not be considered.

## DISCUSSION

This study demonstrates that dermatologist and patient assessments of cosmetic outcome do not necessarily correlate with patient satisfaction. Most respondents expressed negligible concern regarding the resulting scars and instead considered the anticipated effectiveness to be the most important factor when choosing among various available treatment methods for non-facial BCCs. In fact, most patients ranked the cosmetic outcome as the least important factor, below not only the effectiveness but also treatment convenience indicators.

Previous studies have predominantly concentrated on the objective assessments of cosmetic outcomes, using quantitative scar assessment scales, and disregarded the connection to patient satisfaction or their level of concern regarding the aesthetic result (17–19, 25). The absence of this correlation has been noted previously (20). However, one publication comparing imiquimod vs surgery commented on the discrepancy between patient and dermatologist evaluations of the cosmetic outcome, with patients expressing more positive ratings (25).

In addition, earlier comparative studies have either focused on facial lesions or a mix of facial and non-facial lesions. This study exclusively examined non-facial lesions, which we consider valuable, as the majority of low-risk BCCs eligible for non-surgical treatments are non-facial (8). It is therefore important to understand the patients’ preferences for these lesions in order to offer patient-centred and cost-effective treatments. It is nevertheless important to acknowledge that some patients prioritize the cosmetic outcome, also in non-facial areas, such as the lower leg and chest, and therefore a broad palette of treatments is of value. In our study, female sex and age < 60 years were associated with more cosmetic concerns.

BCC guidelines recommend non-surgical treatments as second-line treatments for low-risk lesions (26), which are primarily non-facial lesions, as defined by the National Comprehensive Cancer Network guidelines (27). Topical treatments, including PDT, are often presented as superior alternatives to destructive treatments due to its better cosmesis, but this could be questioned when recurrence rates, costs, and treatment convenience including pain are considered.

In a recent study by Gordon et al., on health-related quality of life in patients with keratinocyte cancer (both facial and non-facial lesions), the lowest impact on quality of life was reported for “appearance” (0.3 on a scale from 0–3), also indicating that the cosmetic concerns are low in general (28). The same study reported slightly higher scores for “worries”, which can imply a greater concern for treatment effectiveness as well as the treatment procedure itself. Also, in a discrete choice study by Essers et al. on topical treatments for superficial BCCs, the anticipated effectiveness of the treatment was the most important factor to the patient (29).

Further, a recent review on cost-conscious treatment approaches for BCCs concluded that for low-risk superficial BCC and nodular BCC, the cost of destructive treatments is 50–60% less compared with PDT or treatment with imiquimod (30). In the same way, Gordon et al. concluded that the costs will be a more important outcome when the cost-effectiveness of interventions is concerned, as keratinocyte cancers’ effect on quality of life and survival may be very small when different treatments are compared (28).

In Sweden, all treatments (including surgery, PDT, destructive treatments, and topical treatments) are covered by tax funding, limiting patient costs to 30 euros per visit, with additional costs for prescription drugs such as imiquimod. When surveyed about cost considerations, half of the patients believed that their own preferences should be prioritized over treatment costs, whereas 31% felt that cost should be a significant consideration. Although not investigated here, patients’ prioritization of cost considerations might differ if they were required to cover a larger proportion of treatment costs themselves. This is an interesting and important area for further research, particularly in countries where patients bear a larger share of treatment costs.

The highest occurrence of BCC is observed in individuals older than 70 years (31), which aligns with the median age of the study population. Some patients mentioned that their perception of scar satisfaction might have differed had they been younger. On the other hand, we have found no research on how patients in different age groups assess the importance of cosmetic outcomes, in relation to other factors, particularly for non-facial lesions. There was a trend towards male patients between 70 and 80 years of age being the most satisfied and the least concerned about scar cosmesis. This may be valuable knowledge as this group constitutes a large proportion of those affected by low-risk non-facial BCCs. There was no significant difference in satisfaction based on the specific type of destructive treatment employed. This finding was surprising, because we initially anticipated that the cosmetic outcome following cryosurgery, which often results in hypopigmentation, would be less favourable compared with curettage alone. However, our results are in line with previous findings by Gordon et al. who reported general low concern about both facial and non-facial lesions.

### Limitations

This study has limitations that should be considered. First, as a single-centre study in Sweden, most patients had skin phototypes I to II, potentially showing less visible scarring than those with darker skin. However, we believe the results are applicable to similar patient groups, in other high-incidence areas in Northern Europe, Australia, and the United States. Second, patients accepting to participate in an RCT on destructive treatments may be less concerned about scarring than the general population.

Third, we adopted a mixed-methods design to enhance our understanding of the quantitative scar assessments and to gain insights into patient treatment preferences. While individual interviews and focus groups are the most common methods for collecting patient experiences, we chose questionnaires to obtain a larger volume of responses, aiming for both quantitative and qualitative data. While this approach limited our ability to ask follow-up questions, it allowed feedback from a broader sample. Finally, since the currently validated scar assessment scales (i.e., 4-point scale and POSAS) did not contain the parameters we wished to study, we developed a new patient and physician rating scale, the ACO scale, which has not been externally validated.

### Conclusion

This study reveals that patients with non-facial BCCs have low concerns about scarring following destructive treatments. Patients with non-facial BCCs prioritize treatment effectiveness, convenience, and time consumption over cosmesis. These findings highlight the need to align treatment decisions with patient preferences, thereby promoting patient-centred care and shared decision-making in the management of BCC, while also taking cost considerations into account.

## Supplementary Material

Patient Preferences and Cosmetic Outcomes Following Destructive Treatments for Non-facial Basal Cell Carcinoma: A Mixed Methods Study

Patient Preferences and Cosmetic Outcomes Following Destructive Treatments for Non-facial Basal Cell Carcinoma: A Mixed Methods Study
