# Supplementary material for: Patient Preferences and Cosmetic Outcomes Following Destructive Treatments for Non-facial Basal Cell Carcinoma: A Mixed Methods Study
Source: Acta Derm Venereol. 2025 Feb 12;105:41325. doi: 10.2340/actadv.v105.41325 (PMC11833252; doi:10.2340/actadv.v105.41325)
Supplement: Patient Preferences and Cosmetic Outcomes Following Destructive Treatments for Non-facial Basal Cell Carcinoma: A Mixed Methods Study [file ActaDV-105-41325-s1.pdf]

Supplementary material has been published as submitted. It has not been copyedited, or typeset by Acta Dermato-Venereologica

APPENDIX S1

	Numeric rating scale (ACO Scale)				
	**1-2**	**3-4**	**5-6**	**7-8**	**9-10**
**Patients**					
Care about scar, *n* (%)	Don't care at all	Don't care much	Neutral	Quite concerned	Very concerned
Satisfaction with scar, *n* (%)	Very satisfied	Moderately satisfied	Neutral	Moderately unsatisfied	Very unsatisfied
Overall scar appearance, *n* (%)	As normal skin	Almost normal	Neither nor	Rather different	Very different
**Dermatologists**					
Overall scar appearance, *n* (%)	As normal skin	Almost normal	Neither nor	Very visible scar	Worst imaginable scar

The numeric rating scale used for the quantitative assessment of cosmetic outcome (ACO) questionnaire.

APPENDIX S2

The questionnaire on patients' perspectives on scars, treatments, preferences and cost (STPC)

This questionnaire aims to deepen our knowledge about how you as a patient experience the scars that resulted from the treatment for basal cell carcinoma in the current study and which factors are important to you regarding the choice of treatment.

Your participation in this survey is voluntary. You have received an invitation to answer this survey because you were included in the research study on destructive treatments of basal cell carcinoma at least three years ago. The goal of this survey is to increase our understanding of the patients' experiences with the scar(s) caused by the destructive treatment as well as how the overall experience of the destructive treatment was. We also endeavor to better understand which factors you consider important when choosing a treatment option. The survey takes about 10-20 minutes to answer. All answers/opinions are valuable to us. If you are unsure about a question or wish to refrain from answering it, you can leave it unanswered.

.

Your experience with the scar(s)

1. Can you describe how you, today, experience the scar(s) that resulted from the destructive treatment of your basal cell carcinoma(s)?

____

2. Based on your description in question 1, what factors influence your perception of your scar(s)?

____

____

____

____

3. Has your experience with the scar(s) changed over time?

Yes ☐ No ☐ Uncertain ☐

4. If you responded yes to question 3, in what way and why do you think this is so?

____

____

____

____

5. Do you feel that you were informed that a scar or a lighter area of skin would appear as a result of the treatment?

Yes ☐ No ☐ Don´t remember ☐

6. If yes, do you think that information matches how it turned out?

____

____

____

Your experience of the treatment

7. What is your overall experience of the treatment(s) you received for your basal cell carcinoma(s)?

____
____
____

8. Do you have experience with other treatments for basal cell carcinoma?

☐ No ☐ Yes, specify which ones…………………………………

9. If you responded yes to question 8, how did you experience the other treatment(s) in comparison to the destructive treatment you received in the study?

____
____
____
____

10. If you responded yes to question 8, what do you think about the scar(s) resulting from the other treatments?

____
____
____
____

Which treatment would you prefer and why?

11. There are alternatives to destructive treatments for a subgroup of basal cell carcinomas, the so-called superficial basal cell carcinomas (which in this study were treated with either scraping only, freezing only or scraping and burning). One option, as a patient, you can apply a prescription cream on the lesion 5 evenings/week for 6 weeks. Another option is a treatment combining a cream and red light, so-called photodynamic therapy, at a Dermatology clinic. This treatment is carried out on two different occasions 1-2 weeks apart and takes about 3.5 hours at each visit. Both of these methods often heal without visible scarring. About 20% of treated basal cell carcinomas recur within 1 year when treated with cream at home and about 25% when treated with photodynamic therapy. With destructive treatment, the risk of recurrence is between 1-5%.
    Would you have preferred either of these two methods to the destructive method given in the study knowing that you could avoid scar formation but have a greater risk of the tumor recurring?

Yes ☐ No ☐

12. Please explain your answer to question 11 here:

____

____

____

____

13. Can you rank the following factors below, based on how important they are to you, regarding the choice of treatment: number **1** being the **most important** factor, number **2** for the **second most important** factor, **3** for the **second least important** and **4** for the **least important**.

☐ The scar cosmesis, i.e., that the resulting scar is as invisible as possible.

☐ That the basal cell carcinoma does not recur after treatment, i.e., that the treatment method is as effective as possible.

☐ Time required for treatment, i.e., if the treatment can be carried out during a first visit to the Dermatology clinic or if two return visits to the hospital are required (approx. 3.5 hours) or if the treatment is carried out at home over a period of 6 weeks.

☐ The time it takes for the wounds to heal.

14. The cost of treating BCC with a destructive method amounts to about 1,500 SEK, for photodynamic therapy about 8,000 SEK and for treatment with a cream at home about 1,500 SEK plus drug costs for the patient of 400-600 SEK. In your opinion, what considerations regarding treatment costs should the tax-financed healthcare system take into account?

☐ None

☐ The patient's own preference should be prioritized over the cost of the treatment.

☐ The cost must be taken into account to a great extent

15. Please add any additional comments and thoughts here:

____

____

____

Thank you for your participation.
