# Supplementary material for: Patient Preferences and Cosmetic Outcomes Following Destructive Treatments for Non-facial Basal Cell Carcinoma: A Mixed Methods Study
Source: Acta Derm Venereol. 2025 Feb 12;105:41325. doi: 10.2340/actadv.v105.41325 (PMC11833252; doi:10.2340/actadv.v105.41325)
Supplement: Patient Preferences and Cosmetic Outcomes Following Destructive Treatments for Non-facial Basal Cell Carcinoma: A Mixed Methods Study [file ActaDV-105-41325-s2.pdf]

Supplementary material has been published as submitted. It has not been copyedited, or typeset by Acta Dermato-Venereologica

Table SI. Patients' overall satisfaction with their scars and how much they care about the scars, stratified by sex and different age groups. Lower scores indicate a more positive evaluation of the scars

	Overall satisfaction with scar					Care about scar				
Variable	NRS score median (range)	NRS score mean (SD)	*n*	Missing data	*P*-value	NRS score median (range)	NRS score mean (SD)	*n*	Missing data	*P*-value
Overall	1 (1-10)	2.2 (1.9)	413	12		1 (1-9)	1.7 (1.5)	413	12	
Males	1 (1-10)	2.0 (1.7)	290	9	0.00005	1 (1-8)	1.46 (1.2)	290	9	0.00001
Females	2 (1-9)	2.6 (2.0)	123	3		1 (1-9)	2 (1,5)	123	3	
Age (years)										
≤ 50	2 (1-9)	2,9 (2.0)	21	0		1 (1-8)	2.1 (1.9)	21	0	
51 – 60	1 (1-10)	3,3 (2.7)	48	1		2 (1-7	2.6 (2.0)	48	1	
61 – 70	1 (1-9)	2,2 (1.9)	83	2	< 0.00001	1 (1-9)	1.6 (1.7)	83	2	< 0.00001
71 – 80	1 (1-9)	1.7 (1.4)	219	1		1 (1-9)	1.5 (1.2)	219	1	
> 80	2 (1-10)	2,6 (1.9)	42	8		2 (1-5)	1.9 (1.0)	42	8	

NRS: Numeric rating scale; SD: standard deviation; *n*: number
